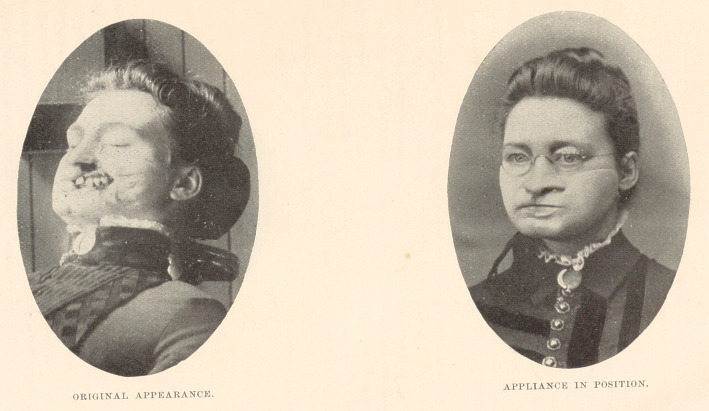# A Delicate Operation Tested by Time

**Published:** 1890-09

**Authors:** Henry N. Dodge

**Affiliations:** Morristown, New Jersey


### A DELICATE OPERATION TESTED BY TIME.

BY HENRY N. DODGE, M.D., D.D.S., MORRISTOWN, NEW JERSEY.

If the case which I wish to describe and record is not unique, it certainly presents features that are rare.

In the spring of 1879, while practising in New York City, a gentleman, aged forty-one, consulted me about his superior incisors, which were crumbling at the cutting edge. This, he said, had been going on since an attack of "nervous prostration" of eight years duration, accompanied by aggravated dyspepsia, acidity of the stomach, and acid saliva.

The patient was then sufficiently recovered to attend to business. Upon examination, I found that not only had the cutting edges of the four incisors been much broken down, but that the enamel was also eroded or dissolved from the entire lingual surfaces of the four incisors (excepting a small part of the right lateral). Most of the lingual surface of the left cuspid was stripped of enamel, and the right cuspid had also suffered from the same cause, but to a slight degree only. These denuded and smoothly-polished surfaces were studded here and there with bosses of gold, the corners of several small proximal fillings of soft gold standing out in bold relief. So soft indeed were these fillings that, had the polishing of the dentine resulted from any form of friction, they would have been worn down long ago to the common level. The impression conveyed to my mind on first examining these teeth was, that the point of the tongue bathed in some solvent had habitually swept over the lingual surfaces of the oral teeth, at first softening and then removing the disintegrated enamel. The idea also suggested itself that lemon-sucking frequently indulged in might have caused such a peculiar result, but this suspicion was groundless, as inquiry proved that the patient had no such habit. On the contrary, alkalies had been long and freely used in the mouth to neutralize the acidity of the saliva. Whatever may have been the etiology of the case, the treatment was as follows: Rubber dam being applied to the incisors and forced well under the gum so as to effectually exclude all moisture, a narrow groove was drilled along the cutting edge of the right central and extended around the lingual surface of the tooth as near as possible to its periphery. Several similar grooves were next cut across the lingual surface parallel with the long axis of the tooth, but not connected with the circumvallating groove.

The grooves were made as deep as circumstances would permit, and were slightly undercut. They were then carefully filled with Williams's cylinders of cohesive gold foil, thoroughly condensed by hand-pressure. When the grooves were all filled, gold was built across from one groove to another until all of the interstices were bridged and the polished surface of dentine entirely covered. This golden surface was then built upon until a sufficient and uniform thickness was attained. The gold upon the cutting edge was carried over a short distance upon the labial surface and also built down sufficiently to give the tooth a somewhat natural outline, but not enough to endanger the stability of the work or to disfigure the mouth with an unpleasant gleam of gold. The gold extended also into those small proximal cavities from which the remains of soft gold fillings had been removed. The whole was then carefully finished, making a strong but delicate armor for the defenceless tooth.

The operation was repeated the next day upon the left central, and at subsequent sittings both laterals were similarly treated. The pulps of these teeth were alive, but that of the left cuspid was dead. The root of this tooth, therefore, after treatment, was filled with gutta-percha, the cavity of decay with gold, and the whole of the lingual surface sheathed with gold in the manner before described. The appearance of the lingual surfaces after treatment is shown in the accompanying figure.

I have recently examined these teeth and replaced some smaller portions of gold about the cutting edges and one corner of a lateral. Once before, since the primary operation, were some very slight additions of gold needed, but, as a whole, the work has remained intact for eleven years. During this time the teeth have been subjected to the severest tests of mastication, the patient being a New York business-man, now in robust health. Time is the crucial test of dentistry, and it has proved the usefulness and permanence of this operation.

## Figures and Tables

**Figure f1:**
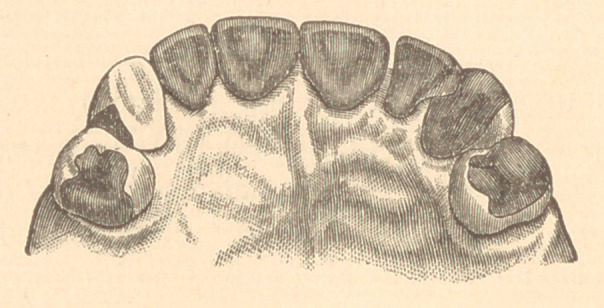


**Figure f2:**